# Evaluation of ecological quality in southeast Chongqing based on modified remote sensing ecological index

**DOI:** 10.1038/s41598-022-19851-9

**Published:** 2022-09-20

**Authors:** Xi Ye, Honghai Kuang

**Affiliations:** School of Geographical Sciences, Southwest University, Chongqing, 400715 China

**Keywords:** Ecosystem ecology, Urban ecology

## Abstract

Rocky desertification is a serious ecological and environmental problem in Southwest China. Quickly and scientifically reveal the distribution and changes of ecological environment quality in rocky desertification areas, which is of great significance to rocky desertification restoration, ecological environment governance and sustainable development. Based on the remote sensing ecological index (RSEI), in addition to greenness, humidity, dryness, and heat, combined with the degree of rocky desertification, this study used the principal component analysis (PCA) method to construct a modified remote sensing ecological index (MRSEI). Then, the temporal and spatial variation characteristics and imaging factors of the ecological environment quality in the typical rocky desertification region of southeastern Chongqing from 2001 to 2021 were explored. The results revealed that the greenness and humidity indicators had a positive effect on the ecological quality, while the indicators of dryness, heat and rocky desertification had the opposite impact. From 2001 to 2021, the ecological environment quality in southeastern Chongqing showed a trend of gradual improvement, and the improvement area accounted for about 70% of the total area. The elevation, slope, monthly average precipitation, and land use pattern were the main factors influencing the quality of the ecological environment in the region.

## Introduction

The quality of the ecological environment can well reflect the comprehensive characteristics of the ecosystem in terms of elements, structure and function^1^. It is closely related to human life and is the foundation of human survival and development^2^. Understanding and evaluating the status of regional ecological environment reasonably is an important way to realize sustainable utilization of natural resources. It is conducive to promoting the harmonious development of social economy and ecological environment, and is vital to the construction of ecological civilization in the country^3–5^.

Rocky desertification is such a serious ecological problem in the subtropical karst regions in southwestern China. Rocky desertification is accompanied by soil erosion, which exposes a large amount of bedrock, degrades the land, and decreases productivity^6^. In addition, due to the intensive human activities, backward economy and culture, and fragile ecological environment in rocky desertification areas, the regional ecology is increasingly degraded. This has become an important obstacle to the sustainable development of the region^7^. Scientific and rapid monitoring of the ecological quality of rocky desertification areas is of great significance to the management of local ecological problems and sustainable development.

The advantage of remote sensing technology is the rapid, real-time, large-scale, periodic and repeated acquisition of comprehensive surface information^8,9^. Therefore, it is widely used in the field of regional ecological quality assessment^10^. A large number of scholars have used remote sensing information to extract a single indicator to investigate the ecological status. For example, vegetation indicators are used to monitor forest ecosystems^11–13^, to study heat island effect by inversion of surface temperature^14–16^, to use water body indicators to extract water body information and conduct ecological evaluation, etc.^17,18^.

The ecological environment is a complex system with many factors acting together. Although the evaluation of a single index has a certain effect, it is difficult to explain its comprehensive effect. Many scholars have begun to try to integrate multiple indicators to assess the quality of ecosystems and their changes. Goward et al. used remote sensing observations to obtain terrestrial soil moisture and combined with surface temperature to evaluate the relationship between soil temperature and soil moisture^19^. MODIS Global Disturbance Index (MGDI) is a collection of Enhanced Vegetation Index (EVI) and Land Surface Temperature (LST), which is mainly used to draw wildfire disturbance maps of a certain scale and regularity^20^. Ochoa-Gaona et al. established an ecological index based on qualitative and semi-quantitative data to measure the ecological status of tropical forests in order to manage and protect tropical forests^21^. Yanchuang et al. found that albedo can monitor changes in dryland ecosystem function by studying the relationship between multiple albedo indicators and the Normalized Difference Vegetation Index (NDVI) and EVI^22^.

Although the current ecological environment quality evaluation has gradually changed from a single-factor evaluation to a multi-index evaluation that combines natural factors and human activity factors. An evaluation system suitable for all evaluation scales and evaluation objects has not yet been formed. The eco-environmental condition index (EI) established from vegetation, organisms, soil, water and human activities has made the eco-environmental quality evaluation standardized to a certain extent^23^. However, this method still faces certain problems. For example, there are many evaluation indicators and the relationship between primary and secondary is difficult to distinguish, and many indicators are not easy to obtain. Some data have a low degree of visualization and are not suitable for small-scale refined research. In addition, the weight setting of each index is sometimes subject to a certain degree of subjectivity and does not take into account the environmental differences within the region. The new remote sensing ecological index (RSEI) which was proposed by Xu^24^ provides a new direction for the evaluation of ecological environment quality. Based on remote sensing technology, RSEI only integrates four easily-obtained ecological indicators of greenness, humidity, heat and dryness, then uses PCA to automatically and objectively assign the weights of each indicator. It cleverly solves the above problems and has been commonly adopted in obtaining ecological quality information^25–29^. In practical applications, many scholars have made various improvements to it according to their needs. For example, in order to make RSEI more comprehensive and representative, some scholars use the first three principal components to construct the MRSEI or combine the entropy weight method with the PCA method to determine the weight of the component indicators^30–32^. According to the ecological environment characteristics of different research areas and based on the RSEI, some scholars have improved the remote sensing ecological index by combining the vegetation net primary productivity (NPP)^33^, salinity index and land degradation index^34^, air quality index^35^, incorporating the human activity intensity index^36^, etc. Considering the nonlinear or weak linear relationship between each index component, some scholars proposed to use the kernel PCA method to construct a nonlinear remote sensing ecological index^35^.

Considering the typical ecological environment problems (rocky desertification phenomenon) in the study area, the degree of rocky desertification is taken as a separate component indicator to better highlight the local ecological environment characteristics. For example, Wang et al. constructed modified remote sensing ecological distance index (MRSEDI) using rocky desertification index (RI), normalized difference built-up and soil index (NDSI), wetness index (WI) and NDVI, and then evaluated the ecological quality in a typical karst area^37^. Therefore, combined with the components of greenness, humidity, dryness and heat, which are commonly used to characterize the quality of the ecological environment, they are introduced into the evaluation system to construct the modified remote sensing ecological index (MRSEI). There is a certain relationship between the indicators in RSEI and rocky desertification, which can reflect the impact of rocky desertification on the quality of the ecological environment. However, RI can directly show the characteristics and changes of rocky desertification, and then directly reveal the impact of rocky desertification characteristics changes on the quality of the ecological environment. The changes and reasons of ecological quality in southeastern Chongqing from 2001 to 2021 were explored through MRSEI. It is hoped that it can provide help for the comprehensive management and scientific development in southeastern Chongqing, and at the same time provide a reference for the ecological quality evaluation in karst areas.

## Materials and methods

### Study area

**Figure 1:**
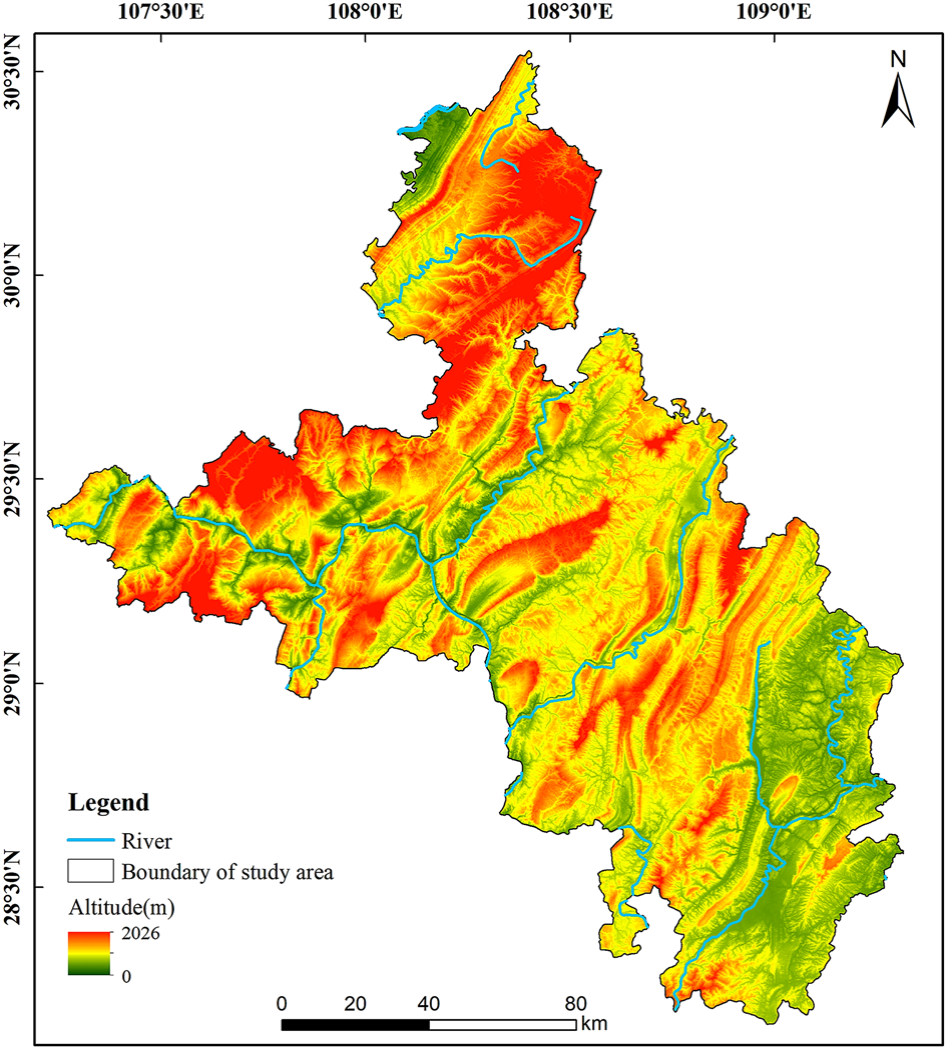
Study area. Map was generated by ArcGIS 10.2 (http://www.esri.com/software/arcgis/arcgis-for-desktop).

Southeastern Chongqing, China (107° 14′–109° 19′ E, 28° 9′–30° 32′ N), has an area of about 19,800 km^2^ (Fig. 1). The study area has a subtropical monsoon climate. And the area has four distinct seasons, with an annual average temperature of 16.2 °C and abundant rainfall, with an average annual rainfall of 1209 mm. This region is located in the central part of the Wuling mountains, which is characterized by medium and low mountainous landforms, with an average altitude of greater than 1000 m. The water system (the Wujiang River system) in the study area is well developed, with a large drainage area and rich groundwater resources. The soil is dominated by yellow soil and limestone soil, and the sensitivity to soil erosion is high. The district exhibits the typical ecological fragility of karst areas, with barren soil, fragmented surfaces, a single community, and a low ecological carrying capacity. The area includes six counties: Qianjiang district, Shizhu Tujia Autonomous county, Xiushan Tujia and Miao Autonomous county, Youyang Tujia and Miao Autonomous county, Wulong district, and Pengshui Miao and Tujia Autonomous county. The coverage rates of the carbonatite layers in these counties are 42.11, 67.77, 25.70, 34.80, 59.70 and 88.46%, respectively^38^, and the average coverage of the carbonatite layers is 53.09%, making this a representative area of karst rocky desertification.

### Data and image pre-processing

In the study, the remote sensing data were obtained from the United States Geological Survey (USGS, https://earthexplorer.usgs.gov/), including landsat-5 thematic mapper (TM) images acquired in 2001, 2006 and 2011 and Landsat-8 operational land imager (OLI) images obtained in 2016 and 2021 (Table 1). The spatial resolution is 30 m. In order to ensure the comparability of spectral characteristics, the data collection was conducted from May to September when the vegetation grew better. In order to meet the usage requirements, the cloud cover of each image used is below 10%. For the images with poor quality, the adjacent years were selected for replacement. The difference in ecological quality between adjacent years in the same region was not particularly large. In order to represent the actual situation of the ecological environment quality in the target year as much as possible, we tried to minimize the replaced part in each target year. A total of 20 images were collected in this study. The images downloaded were all L1T products, which had undergone systematic radiometric correction and geometric correction, so precise geometric correction was no longer performed. Before the subsequent processing, all 20 images were preprocessed by radiometric calibration, atmospheric correction, image mosaicking and cropping. Then these images were calculated to obtain NDVI, WET, NDBSI, LST and RI. And based on the preprocessed Landsat images, support vector machine classification was performed to obtain the land use (LU) status.

**Table 1:** Information of images used in this study.

Path/row	2001 (Landsat TM)		2006 (Landsat TM)		2011 (Landsat TM)		2016 (Landsat OLI)		2021 (Landsat OLI)	
	Date	Cloud (%)	Date	Cloud (%)	Date	Cloud (%)	Date	Cloud (%)	Date	Cloud (%)
126/39	2000/9/2	5.00	2006/8/2	5.00	2011/9/1	2.00	2016/7/28	0.09	2020/6/5	7.59
126/40	2001/9/5	5.00	2005/7/30	10.00	2011/8/16	4.00	2016/8/29	3.26	2020/6/5	5.05
127/39	2001/7/26	6.00	2006/8/9	9.00	2011/5/19	1.00	2016/6/17	0.25	2021/8/2	1.55
127/40	2002/6/11	2.00	2005/6/3	8.00	2011/5/19	0.00	2016/6/17	5.36	2021/8/2	0.75

The topographical data included the elevation (EV) and slope (SP) data. Among them, the elevation data was provided by the official website of the United States Geological Survey (USGS, https://earthexplorer.usgs.gov/). And the slope data was calculated from the elevation data. The meteorological data, including the monthly average temperature (MT), monthly mean precipitation (PR), monthly even relative humidity (RH), and monthly total sunshine hours (SH) from May to September of the target year, were got from the China Meteorological Data Network (http://data.cma.cn/). In addition, socioeconomic data, including the population density (PD) and gross domestic product (GDP), were obtained from the statistical yearbooks of each district and county in the study area. The nighttime light (NTL) data were obtained from the National Oceanic and Atmospheric Administration (NOAA, https://www.noaa.gov/). The above data and LU were used as the influencing factors of ecological quality to analyze the reasons for the change of local ecological environment quality. The statistical data and monitoring data of each evaluation index used to construct the EI come from the statistical yearbooks, water resources bulletin and soil and water conservation bulletin of each district and county.

### Methodology

#### Study framework

**Figure 2:**
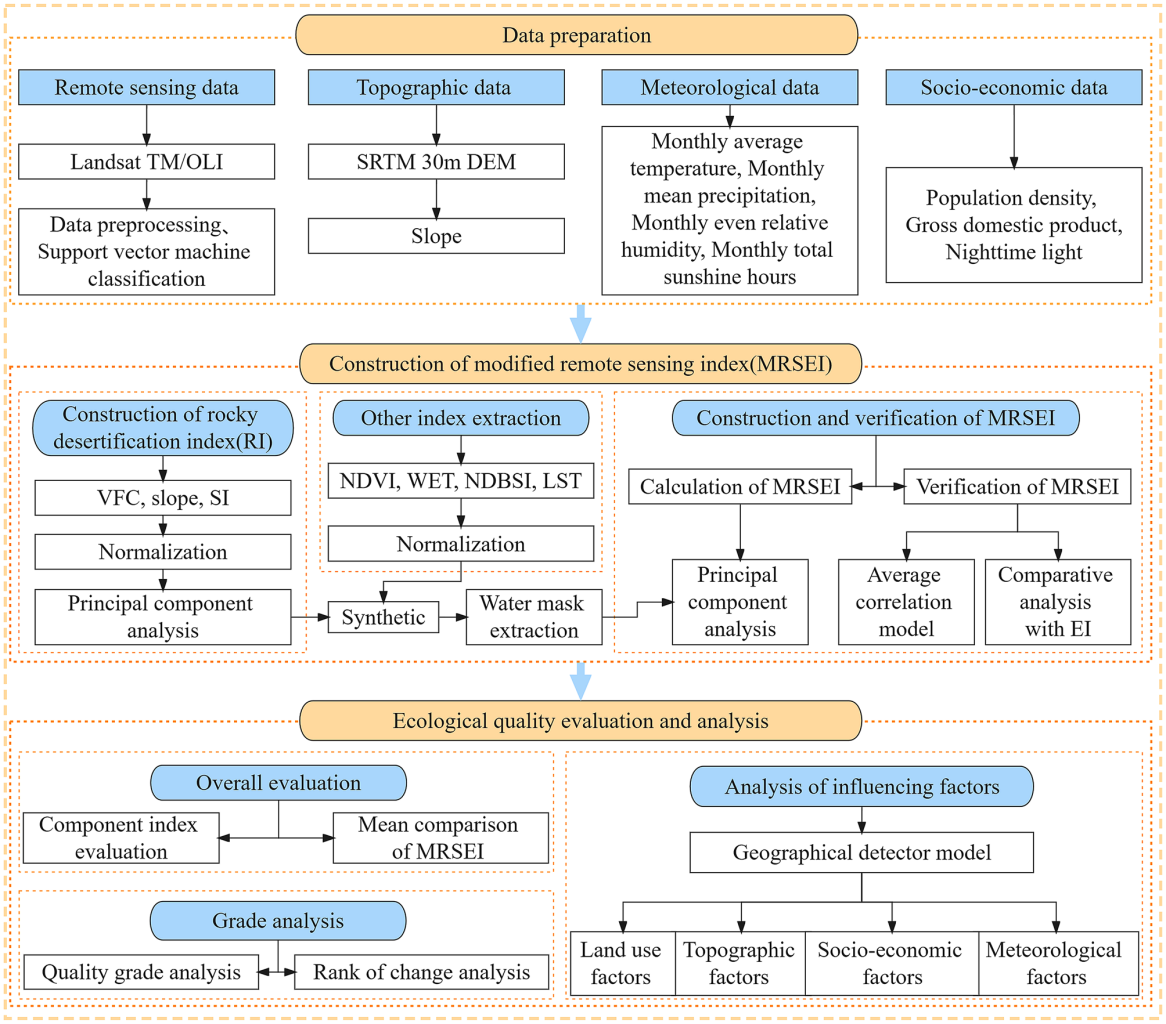
The study framework.

A framework was developed for evaluating the ecological quality in southeastern Chongqing from 2001 to 2021 in the study. And the framework included three parts: data preparation, construction of the MRSEI, and the analysis of the ecological status in the region. Figure 2 presents the detailed information about the framework. The operations of band calculation, normalization and PCA were all carried out using the ENVI 5.3 software (https://www.harrisgeospatial.com).

#### Indicators used in MRSEI

The greenness, humidity, heat, dryness, and degree of rocky desertification were used to construct the MRSEI. The NDVI^39^ was chosen to characterize the greenness. The humidity component acquired from the tasseled cap transformation (WET)^40^ was selected to represent the humidity. The LST^41^ was used to represent the heat, the normalized difference build-up soil index (NDBSI)^42^ was used to characterize the dryness. The RI was applied to characterize the degree of rocky desertification.

The NDVI is an important indicator for monitoring the physical and chemical properties of vegetation, and it can be employed to calculate the vegetation coverage, leaf area index, and so on^19^. In addition, it eliminates some radiation errors and has a stronger response to surface vegetation. It has been widely used in vegetation remote sensing monitoring. The equation for calculating the NDVI is as follows^39^:

1 $$ {\text{NDVI}} = {{(\uprho }}_{{{\text{NIR}}}} - {\uprho }_{{{\text{Red}}}} {)}/{{(\uprho }}_{{{\text{NIR}}}} {{ + \uprho }}_{{{\text{Red}}}} ), $$

where ${\uprho }_{{{\text{NIR}}}}$ is the reflectance of the near-infrared band and ${\uprho }_{{{\text{Red}}}}$ refers to the reflectance of the red band corresponding to each image.

The WET can effectively reflect the humidity conditions of the surface vegetation, water, and soil, and can reveal the changes in the ecological environment, such as soil degradation. Therefore, it is commonly used in ecological environment monitoring^43^. The WET can be expressed as^40,43^:

2 $$ {\text{WET}}_{{{\text{TM}}}} { = 0}{{.3102\uprho }}_{{{\text{Red}}}} { + 0}{{.2021\uprho }}_{{{\text{Green}}}} { + 0}{{.0315\uprho }}_{{{\text{Blue}}}} { + 0}{{.1594\uprho }}_{{{\text{NIR}}}} - {0}{{.6806\uprho }}_{{{\text{SWIR1}}}} - {0}{{.6109\uprho }}_{{{\text{SWIR2}}}} , $$

3 $$ {\text{WET}}_{{{\text{OLI}}}} { = 0}{{.3283\uprho }}_{{{\text{Red}}}} { + 0}{{.1972\uprho }}_{{{\text{Green}}}} { + 0}{{.1511\uprho }}_{{{\text{Blue}}}} { + 0}{{.3407\uprho }}_{{{\text{NIR}}}} - {0}{{.7117\uprho }}_{{{\text{SWIR1}}}} - {0}{{.4559\uprho }}_{{{\text{SWIR2}}}} , $$

where ${\uprho }_{{\text{i}}} \,$ is the reflectance of band i.

The NDBSI is expressed as the average of two indicators, the bare soil index (SI)^44^ and the index-based built-up index (IBI)^45^. It can be applied to characterize the dryness. The calculation formulas are^44,45^:

4 $$  {\text{IBI }} = {\text{ }}\left[ {2\uprho _{{{\text{SWIR1}}}} /\left( {\uprho _{{{\text{SWIR1}}}}  + {\text{ }}\uprho _{{{\text{NIR}}}} } \right) - \uprho _{{{\text{NIR}}}} /(\uprho _{{{\text{NIR}}}}  + {\text{ }}\uprho _{{{\text{Red}}}} } \right) - \uprho _{{{\text{Green}}}} /(\uprho _{{{\text{Green}}}}  + {\text{ }}\uprho _{{{\text{SWIR1}}}} )]/[2\uprho _{{{\text{SWIR1}}}} /\left( {\uprho _{{{\text{SWIR1}}}}  + {\text{ }}\uprho _{{{\text{NIR}}}} } \right) + {\text{ }}\uprho _{{{\text{NIR}}}} /(\uprho _{{{\text{NIR}}}}  + {\text{ }}\uprho _{{{\text{Red}}}} ) + {\text{ }}\uprho _{{{\text{Green}}}} /(\uprho _{{{\text{Green}}}}  + {\text{ }}\uprho _{{{\text{SWIR1}}}} )], $$

5 $$ {\text{SI = }}\left[ {{\uprho }_{{{\text{SWIR1}}}} {{ + \uprho }}_{{{\text{red}}}} - \left( {{\uprho }_{{{\text{Blue}}}} {{ + \uprho }}_{{{\text{NIR}}}} } \right)} \right]/\left[ {{\uprho }_{{{\text{SWIR1}}}} {{ + \uprho }}_{{{\text{red}}}} { + }\left( {{\uprho }_{{{\text{Blue}}}} {{ + \uprho }}_{{{\text{NIR}}}} } \right)} \right], $$

6 $$ {\text{NDBSI = (IBI + SI)/2,}} $$

where ${\uprho }_{{\text{i}}} \,$ is the reflectance of band i.

The LST is closely related to natural processes and human phenomena such as crop yield, vegetation growth and distribution, surface water cycle, etc. It can well reflect the state of the surface ecological environment. The atmospheric correction method is used to invert the LST here^46,47^, it can be expressed as:

7 $$ {\text{L = gain}} \times {\text{DN + bias,}} $$

8 $$ {\text{T = K}}_{{2}} /{\text{ln}}\left( {\frac{{{\text{K}}_{{1}} }}{{\text{L}}}{ + 1}} \right){,} $$

9 $$ {\text{LST = T}}/\left[ {{1 + }\left( {\frac{{{\lambda T}}}{{\upalpha }}} \right){{ln\varepsilon }}} \right]{,} $$

where L is the radiation value in the thermal infrared band, DN is the gray value, gain and bias is the gain value and offset value of the L-band, which was got from the image header file. And T is the temperature value at the sensor; K1 and K2 are calibration parameters respectively (for TM, K1 = 607.76 W/(m^2^ sr μm), K2 = 1260.56 K; for TIRS, K1 = 774.89 W/(m^2^ sr μm), K2 = 1321.08 K); λ is the central wavelength of thermal infrared band; α = 1.438 × 10^−2^ m K. ε is the surface emissivity and the value is estimated by the vegetation index mixture model^48,49^. It is calculated as follows:

10 $$ {\text{VFC = }}\frac{{{\text{NDVI}} - {\text{NDVI}}_{{{\text{Soil}}}} }}{{{\text{NDVI}}_{{{\text{Veg}}}} - {\text{NDVI}}_{{{\text{Soil}}}} }}, $$

11 $$ {\text{d}}_{{\upvarepsilon }} { = }\left( {{1} - {\upvarepsilon }_{{\text{s}}} } \right){{ \times (1}} - {\text{VFC) }}\times \text{F} \times \upvarepsilon _{{\text{v}}} , $$

12 $$ {{\upvarepsilon = \upvarepsilon }}_{{\text{v}}} \times {\text{ VFC}} + \varepsilon _{{\text{s}}} {{ \times }}\left( {{1} - {\text{FVC}}} \right){\text{ + d}}_{{\upvarepsilon }} , $$

where VFC is the vegetation fractional cover, ${\text{NDVI}}_{{{\text{Veg}}}}$ is the NDVI of the pixel covered by full vegetation and the pixels with NDVI > 0.72 are regarded as pure vegetation pixels; ${\text{NDVI}}_{{{\text{Soil}}}}$ is the NDVI of the bare pixel and the pixels with NDVI < 0.10 are regarded as pure bare soil pixels. Pixels with 0.10 ≤ NDVI ≤ 0.72 are regarded as mixed pixels, Calculated by Eq. (12). ${\text{d}}_{{\upvarepsilon }}$ is the error caused by terrain relief and satellite tilt observations ${\upvarepsilon }_{{\text{v}}}$ is the radiance of pure vegetation pixels, and its value is 0.985, ${\upvarepsilon }_{{\text{s}}}$ is the radiance of pure bare soil pixels, and its value is 0.960, F is the terrain factor, generally 0.55.

At present, there is no definite rocky desertification evaluation system, and remote sensing rocky desertification monitoring needs to consider the characteristics of the remote sensing technology and the application requirements for the rocky desertification evaluation^50^. The bedrock exposure (Fr) and VFC are the most intuitive ground performance features of rocky desertification, and they are the key indicators for rocky desertification evaluation^51^. The slope affects the soil erosion, which is one of the reasons for rocky desertification^52^. Therefore, considering the geographical conditions of the study area and the availability of data, combined with previous research results^53,54^, VFC, Fr and slope were selected as evaluation indicators. The PCA was performed on these indicators to construct the RI. The slope can be obtained by calculation from the DEM. The vegetation coverage was estimated based on NDVI by the pixel dichotomy method^55^. The normalized difference rock index (NDRI) was used to estimate the exposure of bedrock by pixel dichotomy^56^. The VFC is calculated by Eq. (12), the NDVI with a 5% cumulative frequency was selected as the NDVI for the bare soil pixels, while the NDVI with a 95% cumulative frequency was chosen as the NDVI for the full vegetation coverage pixels here^55^.

The expression for NDRI is^56^:

13 $$ {\text{NDRI}} = \uprho _{{{\text{SWIR}}}} - {\uprho }_{{{\text{NIR}}}} {{/(\uprho }}_{{{\text{SWIR}}}} {{ + \uprho }}_{{{\text{NIR}}}} {),} $$

where ${\uprho }_{{{\text{SWIR}}}}$ and $\uprho_{NIR}$ represent the reflectivity of the short-wave infrared band and the near-infrared band corresponding to each image, respectively.

And the expression for Fr is^56^:

14 $$ {\text{Fr = NDRI}} - {\text{NDRI}}_{{{\text{Rock}}}} {\text{/(NDRI}}_{{{\text{Rock}}}} - {\text{NDRI}}_{{0}} {),} $$

where ${\text{NDRI}}_{{{\text{Rock}}}}$ is the NDRI of the pixel with full rock coverage, and ${\text{NDRI}}_{0}$ is the NDVI of the pixel without rock coverage. In the study, the values of ${\text{NDRI}}_{{{\text{Rock}}}}$ and ${\text{NDRI}}_{{0}}$ are the maximum and minimum values within the confidence interval of 95% confidence in the image, respectively.

Due to the different units of the three indicators used to establish the RI, for the sake of facilitating comparison and calculation and to eliminate the weight imbalance caused by their different units, the three indicators were normalized to a range of 0–1. The expression for the normalized value of the indicators is:

15 $$ {\text{NI = }}\frac{{{\text{I}} - {\text{I}}_{{{\text{min}}}} }}{{{\text{I}}_{{{\text{max}}}} - {\text{I}}_{{{\text{min}}}} }}, $$

where ${\text{I}}$ is the value for a pixel, ${\text{I}}_{{{\text{max}}}}$ and ${\text{I}}_{{{\text{min}}}}$ represent the maximum and minimum values of the pixel, respectively.

The band synthesis and PCA of the three indexes after normalization was carried out, and then, the initially RI (RI_0_) was obtained as follows:

16 $$ {\text{RI}}_{{0}} {\text{ = PC}}_{{1}} {\text{[f(SI, VFC, SP}})]. $$

In order to make the RI correspond to the degree of rocky desertification, for the negative correlations, ${\text{RI}}_{0}$ was acquired using the following formula:

17 $$ {\text{RI}}_{{0}} { = 1} - {\text{PC}}_{{1}} {\text{[f(SI, VFC, SP)]}}{.} $$

In addition, RI is obtained by normalizing ${\text{RI}}_{{0}}$ by formula 15, which is convenient for analysis and comparison between different years.

#### Calculation of MRSEI

The five component metrics (NDVI, WET, LST, NDBSI, and RI) were normalized to eliminate the dimensional difference, and then band synthesis was carried out. The water system in the study area is well developed. The water area was extracted using the modified normalized difference water index (MNDWI)^57^ to prevent large-scale water from affecting the reflection of the humidity indicators. Then, it was removed by applying the mask. PCA was performed on the synthetic images after removing the water range, and the initial MRSEI (${\text{MRSEI}}_{0}$) was constructed. And the equation for this index is:

18 $$ {\text{MRSEI}}_{{0}} {\text{ = PC}}_{{1}} {\text{[f(NDVI,}}\,{\text{WET,}}\,{\text{NDBSI,}}\,{\text{LST,}}\,{\text{RI)],}} $$

where ${\text{PC}}_{{1}}$ is the first principal component obtained by PCA. In addition, for the negative correlation results, a positive and negative transposition operation was needed. The initial modified remote sensing ecological index is:

19 $$ {\text{MRSEI}}_{{0}} { = 1} - {\text{PC}}_{{1}} {\text{[f(NDVI,}}\,{\text{WET,}}\,{\text{NDBSI,}}\,{\text{LST,}}\,{\text{RI}})]. $$

Similarly, it was necessary to normalize the ${\text{MRSEI}}_{0}$ to obtain the MRSEI for eliminating the influence of dimension. And the quality of the ecological environment was proportional to the size of its value. The larger the MRSEI, the better the ecological quality.

#### Comprehensiveness verification of MRSEI

The correlation degree was used to determine the comprehensive representative degree of the MRSEI. Its value is between 0 and 1, and the larger the value, the better the comprehensive representation of MRSEI^58^. The formula is:

20 $$ {\overline{\text{C}}}_{{\text{p}}} { = }\frac{{{\text{(|C}}_{{\text{q}}} {\text{| + |C}}_{{\text{r}}} {| + } \cdots {\text{| C}}_{{\text{s}}} {|)}}}{{{\text{n}} - {1}}}, $$

where ${\overline{\text{C}}}$_p_ is the average correlation; p, q, r and s are the indicators for the correlation analysis; ${\text{C}}_{{\text{q}}}$, ${\text{C}}_{{\text{r}}}$, and ${\text{C}}_{{\text{s}}}$ are the correlation coefficients between p and q, r and s during the same period, respectively, and n is the number of indicators for the correlation analysis.

#### Geographical detector method

The geographical detector aims to probing spatial heterogeneity and uncovering its causes. It can not only analyze the relationship between a single independent variable and a dependent variable, but it can also analyze the interactions between two independent variables. In addition, it can accurately determine the strength, direction, and linear or nonlinear relationship between the two variables, and thus, it can reflect the driving force behind the dependent variable to solve practical problems^59,60^. The factor detector is mainly applied to detect the spatial differentiation of the dependent variables and the degree of interpretation of the independent variables to the spatial heterogeneity of the dependent variables. The degree of explanation of the independent variable corresponding to the variable is expressed by the q value^61–63^. In this study, the factor detector was mainly provided to analyze the influences of the various factors on the spatial differentiation of the quality of the ecological environment in southeastern Chongqing. And it can be expressed as:

21 $$ {\text{q = 1}} - \frac{{\mathop \sum \nolimits_{{\text{h = 1}}}^{{\text{L}}} {\text{N}}_{{\text{h}}} {\upsigma }_{{\text{h}}}^{{2}} }}{{{\text{N}}\upsigma ^{{2}} }}, $$

where q is the influence of the affecting factors on the spatial heterogeneity of the ecological status, ${\upsigma }^{{2}}$ is the variance of the MRSEI in the entire region, ${\upsigma }_{{\text{h}}}^{{2}}$ is the variance of the MRSEI in sub-level region h, N is the total number of samples in the region, ${\text{N}}_{{\text{h}}}$ is the number of sub-level regional samples, and L is the total number of sub-level regions.

#### Construction of EI

According to *Technical Specifications for Evaluation of Ecological Environment Status (HJ 192-2015)*^23^, five sub-index and 20 sub-indicators were selected to construct EI. Taking the sub-indicators as the basic data, the sub-index was obtained by calculation, and then the EI is obtained by weighted calculation of the sub-index. The expression for EI is as follows:

22 $$ {\text{EI = 0}}{{.35 \times HQI + 0}}{{.25 \times VCI + 0}}{{.15 \times WNDI + 0}}{{.15 \times }}\left( {{100} - {\text{LSI}}} \right){ + 0}{{.1 \times (100}} - {\text{PLI),}} $$

where HOI is the Habitat Quality Index, VCI is the Vegetation Cover Index, WND is the Water Network Density Index, LSI is the Land Stress Index, PLI is the Pollution Load Index. The weights of the sub-indicators are determined by AHP, and they are shown in Table 2.

**Table 2:** EI construction indicators and their weights.

Comprehensive index	Sub-index	Index weight	Sub-indicators	Weight of sub-indicator
EI	HQI	0.35	Arableland	0.11
			Woodland	0.35
			Grassland	0.21
			Water body and wetlands	0.28
			Construction land	0.04
			Unused	0.01
	VCI	0.25	NDVI	–
	WNDI	0.15	Length of river	–
			Area of water	–
			Amount of water resources	–
	LSI	0.15	Heavy erosion	0.40
			Moderate erosion	0.20
			Construction land	0.20
			Other land	0.20
	PLI	0.10	Cod	0.20
			Ammonia nitrogen	0.20
			SO_2_	0.20
			Soot and dust	0.10
			Nitrogen oxides	0.20
			Solid Waste	0.10

## Results

### Validation results of the mean correlation model

The applicability of the MRSEI was tested using the average correlation model. The correlation coefficients between each component index and the MRSEI and between each index were shown in Table 3. In terms of sub-indexes, the average correlation of the dryness index was higher than those of the other sub-indexes in 2001, 2011, 2016, and 2021, with an average of 0.663 during the five periods. The average correlation of the RI (0.506) was higher in 2006 than those of the other sub-indexes. The lowest average correlation index was the heat index, with an average of 0.395 during the five periods. The correlation coefficients between the MRSEI and the component indexes were greater than 0.631 during each period, and the average value during the five periods reached 0.753. Therefore, there was a good correlation between the MRSEI and each component index, which was more representative than any single index and can better reflect the regional ecological quality.

**Table 3:** Statistics of the correlations between the MRSEI and the component indicators.

Period	Index	LST	NDBSI	NDVI	RI	WET	MRSEI
2001	LST	1.000	0.339	− 0.338	0.356	− 0.254	− 0.444
	NDBSI	0.339	1.000	− 0.631	0.688	− 0.745	− 0.730
	NDVI	− 0.338	− 0.631	1.000	− 0.954	0.284	0.952
	RI	0.356	0.688	− 0.954	1.000	− 0.306	− 0.994
	WET	− 0.254	− 0.745	0.284	− 0.306	1.000	0.365
	$\overline{{\text{C}}}$	0.322	0.601	0.552	0.576	0.397	0.697
2006	LST	1.000	0.373	− 0.193	0.279	− 0.188	− 0.335
	NDBSI	0.373	1.000	− 0.372	0.604	− 0.418	− 0.626
	NDVI	− 0.193	− 0.372	1.000	− 0.897	0.376	0.905
	RI	0.279	0.604	− 0.897	1.000	− 0.244	− 0.997
	WET	− 0.188	− 0.418	0.376	− 0.244	1.000	0.291
	$\overline{{\text{C}}}$	0.258	0.442	0.460	0.506	0.306	0.631
2011	LST	1.000	0.531	− 0.472	0.492	− 0.337	− 0.605
	NDBSI	0.531	1.000	− 0.753	0.807	− 0.643	− 0.823
	NDVI	− 0.472	− 0.753	1.000	− 0.954	0.490	0.951
	RI	0.492	0.807	− 0.954	1.000	− 0.438	− 0.991
	WET	− 0.337	− 0.643	0.490	− 0.438	1.000	0.466
	$\overline{{\text{C}}}$	0.458	0.683	0.667	0.673	0.477	0.767
2016	LST	1.000	0.467	− 0.431	0.442	− 0.386	− 0.573
	NDBSI	0.467	1.000	− 0.872	0.889	− 0.895	− 0.908
	NDVI	− 0.431	− 0.872	1.000	−0.940	0.659	0.937
	RI	0.442	0.889	− 0.940	1.000	− 0.696	− 0.988
	WET	− 0.386	− 0.895	0.659	− 0.696	1.000	0.724
	$\overline{\mathrm{C} }$	0.432	0.781	0.725	0.742	0.659	0.826
2021	LST	1.000	0.539	− 0.511	0.517	− 0.459	− 0.615
	NDBSI	0.539	1.000	− 0.880	0.893	− 0.924	− 0.915
	NDVI	− 0.511	− 0.880	1.000	− 0.948	0.695	0.946
	RI	0.517	0.893	− 0.948	1.000	− 0.736	− 0.992
	WET	− 0.459	− 0.924	0.695	− 0.736	1.000	0.766
	$\overline{{\text{C}}}$	0.507	0.809	0.758	0.773	0.704	0.847
Mean value of $\overline{{\text{C}}}$		M_LST	M_NDBSI	M_NDVI	M_RI	M_WET	M_MRSEI
		0.395	0.663	0.634	0.654	0.509	0.753

### Variation characteristics of five indices

**Figure 3:**
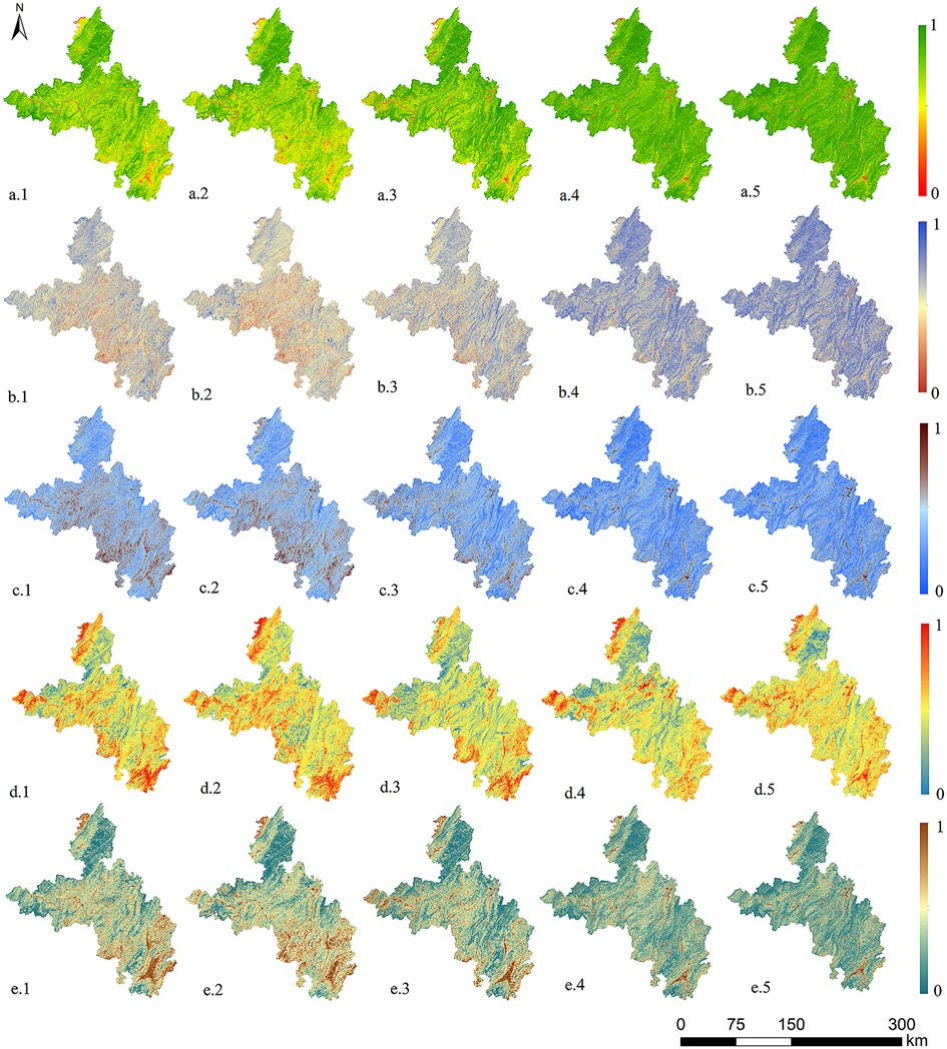
Distributions of the five indices: (**a.1–5**) NDVI, (**b.1–5**) WET, (**c.1–5**) NDBSI, (**d.1–5**) LST and (**e.1–5**) RI in 2001, 2006, 2011, 2016 and 2021 (1–5 from left to right). Map was generated by ArcGIS 10.2 (http://www.esri.com/software/arcgis/arcgis-for-desktop).

The changes in the components of each indicator during these five periods can be learn from Fig. 3. From 2001 to 2006, the vegetation coverage in the outer edge of the northeastern region and the western edge was relatively good, but it was poor in the southern part, and the overall change was small. In the following 10 years, the NDVI exhibited an upward trend. From 2016 to 2021, the overall distribution of the NDVI did not change significantly. The WET initially decreased and then gradually increased. During the first period, there is an obvious drop in the northeastern, central, southern parts. And then it significantly increased from 2006 to 2021, but the increase gradually slowed. From 2001 to 2016, the NDBSI gradually decreased, and it significantly decreased in the southern and central parts. During the last period, the NDBSI increased slightly, and the overall distribution did not change significantly. In the last two decades, the fluctuation range of the LST was small, and the high temperature areas were concentrated in the western, the western margin of the northeast, and the southern parts. In the first 5 years, the RI increased briefly, and the areas with more severe rocky desertification were concentrated in the western margin of the northeast, the central, and the southern parts. In the later time, the degree of rocky desertification gradually decreased.

### Ecological status of the study area

The eigenvalue contribution rate of PC1 in 2001, 2006, 2011, 2016, and 2021 after principal component transformation was > 80%, showing that PC1 is sufficient to represent the comprehensive information (Table 4). And the indicators make a certain contribution to PC1, the load signs of NDVI, WET and LST, NDBSI, RI were opposite (Table 5). It meant that the above two sets of indicators had opposite effects on the ecology. According to a previous study^17^, the NDVI and WET had positive effects on the ecological quality while the LST, NDBSI, and RI had negative impacts. In 2001, 2011, and 2021, the load signs of NDVI and WET were positive, and the load signs of LST, NDBSI, and RI were negative. Thus, the MRSEI constructed in these periods was positively correlated with the ecological quality. The situations in 2006 and 2016 were the exact opposite of the above 3 years. The results obtained in 2006 and 2016 were negatively correlated. The larger the value of MRSEI, the worse the quality of the ecological environment. For the convenience of comparison, the positive and negative transposition of the PCA results in 2006 and 2016 was carried out, so that the areas with better ecological environment quality had higher MRSEI values.

**Table 4:** Principal component analysis results.

Period	Types	PC1	PC2	PC3	PC4	PC5
2001	Eigenvalue	0.3952	0.0386	0.0271	0.0074	0.0013
	Contribution (%)	80.900	10.630	5.270	2.800	0.400
2006	Eigenvalue	0.4251	0.0514	0.0327	0.0080	0.0025
	Contribution (%)	83.300	14.770	1.470	0.380	0.080
2011	Eigenvalue	0.3530	0.0562	0.0347	0.0085	0.0019
	Contribution (%)	81.080	16.990	1.490	0.350	0.090
2016	Eigenvalue	0.3418	0.0273	0.0216	0.0072	0.0015
	Contribution (%)	85.830	12.260	1.740	0.130	0.040
2021	Eigenvalue	0.4164	0.0443	0.0268	0.015	0.0022
	Contribution (%)	80.530	12.080	6.160	0.790	0.440

PC1, PC2, PC3, PC4, PC5 represent the first to fifth principal components, respectively.

**Table 5:** Mean and PC1 load statistics of the five indicators.

Period	Types	LST	NDBSI	NDVI	RI	WET	MRSEI
2001	Mean	0.681	0.505	0.746	0.457	0.545	0.561
	PC1 Load	− 0.116	− 0.291	0.318	− 0.402	0.184	
2006	Mean	0.631	0.481	0.743	0.468	0.448	0.574
	PC1 Load	0.166	0.224	− 0.327	0.474	− 0.132	
2011	Mean	0.529	0.448	0.803	0.415	0.670	0.632
	PC1 Load	− 0.171	− 0.143	0.352	− 0.349	0.204	
2016	Mean	0.547	0.404	0.874	0.364	0.785	0.659
	PC1 Load	0.187	0.127	− 0.361	0.298	− 0.267	
2021	Mean	0.556	0.422	0.868	0.333	0.798	0.678
	PC1 Load	− 0.233	− 0.181	0.407	− 0.298	0.274	

Table 5 shows that from 2001 to 2021, the mean MRSEI in the study area increased from 0.561 in 2001 to 0.678 in 2021 (by 20.9%), the ecological status gradually improved. The NDVI and the WET, which promoted the ecological environment, increased by 16.4 and 46.4%, respectively, in the last two decades. The heat index decreased by 18.3% at the end of this entire period. The decrease in the NDBSI was also distinct, with a decrease of 14.5%. With the vigorous development of rocky desertification control in the study area since 2006, the RI also significant decreased (by 27.1%). There was a significant improvement in local habitat quality.

**Figure 4:**
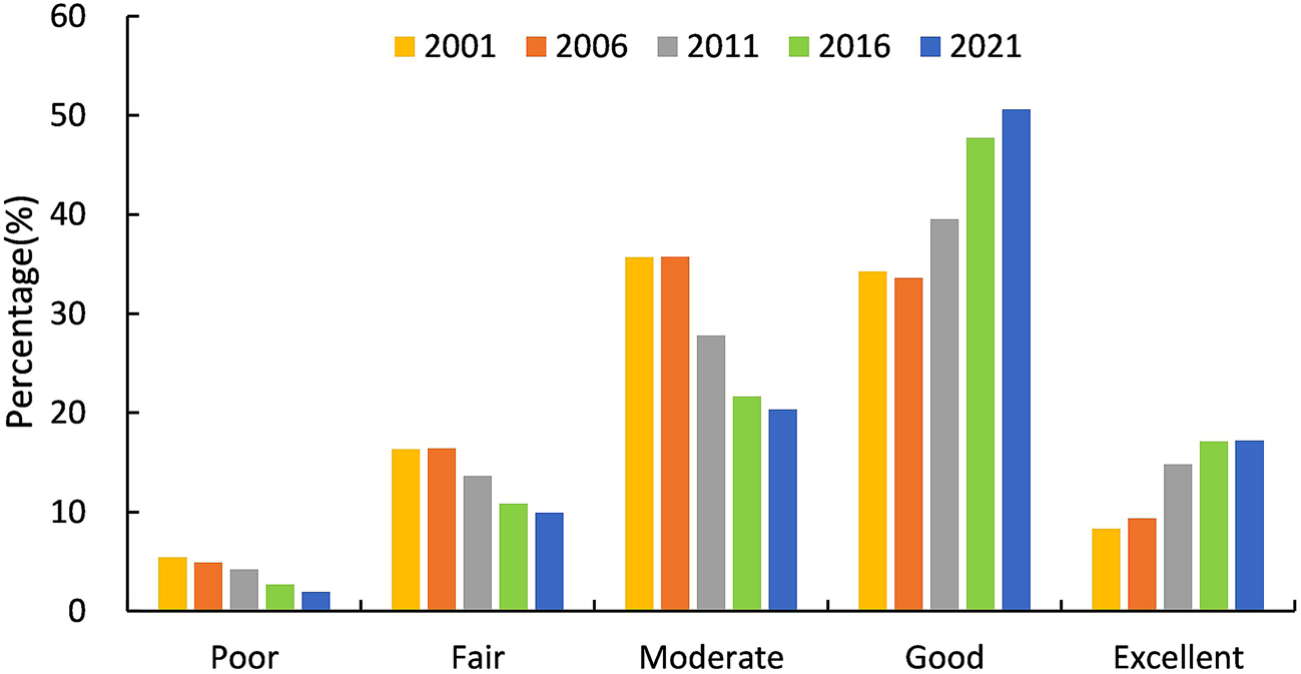
Percentage of each MRSEI grade.

According to previous studies, the equal interval method was adopted, and 0.2 was used as the interval. The MRSEI was divided into five grades (poor, fair, moderate, good and excellent) to analyze the distribution and changes of the ecological status. During the past two decades, the area and proportion of the ecological quality grades are shown in Table 6 and Fig. 4.

**Table 6:** Area and percentage of each MRSEI grade.

Level	2001		2006			2011			2016			2021	
	Area	Pct	Area	Pct	Area		Pct	Area		Pct	Area		Pct
	(km^2^)	(%)	(km^2^)	(%)	(km^2^)		(%)	(km^2^)		(%)	(km^2^)		(%)
Poor	1035.337	5.421	912.875	4.892	801.566		4.223	510.854		2.694	356.837		1.928
Fair	3117.471	16.325	3124.694	16.433	2587.044		13.621	2052.442		10.824	1892.374		9.903
Moderate	6817.557	35.692	6797.112	35.741	5282.357		27.805	4105.037		21.648	3862.568		20.361
Good	6540.576	34.243	6403.435	33.672	7506.607		39.524	9049.740		47.724	9606.122		50.604
Excellent	1591.209	8.332	1780.105	9.352	2816.877		14.828	3244.321		17.109	3262.779		17.202

It can be seen that in 2001 and 2006, the proportions of the ecological grades were basically similar, and the major grades were moderate and good, accounting for nearly 70% of the whole region. The excellent ecological quality areas accounted for 8.33% in 2001 and 9.35% in 2006, respectively, with an increase in 2006 compared with 2001. By 2011, the ecological quality had significantly improved, the good and excellent quality areas had distinctly increased. Compared with 2006, the area with a good ecological grade increased by 1103.172 km^2^, the area with an excellent ecological quality increased by 1036.772 km^2^. In 2016, the condition of the ecological environment continued to improve, the poor ecological quality areas decreased by 1.53% in this time. In addition, the proportion of the area of good and excellent grade in ecological quality promoted prominently. In 2021, the conditions continued to improve. The proportion of the area with good ecological quality grade exceeds 50% of the entire region while the area of poor ecological quality grade decreased by 154.017 km^2^ compared with 2016. The MRSEI continuously increased from 2001 to 2021, demonstrating that the ecological status of the ecological in southeastern Chongqing had gradually improved.

**Figure 5:**
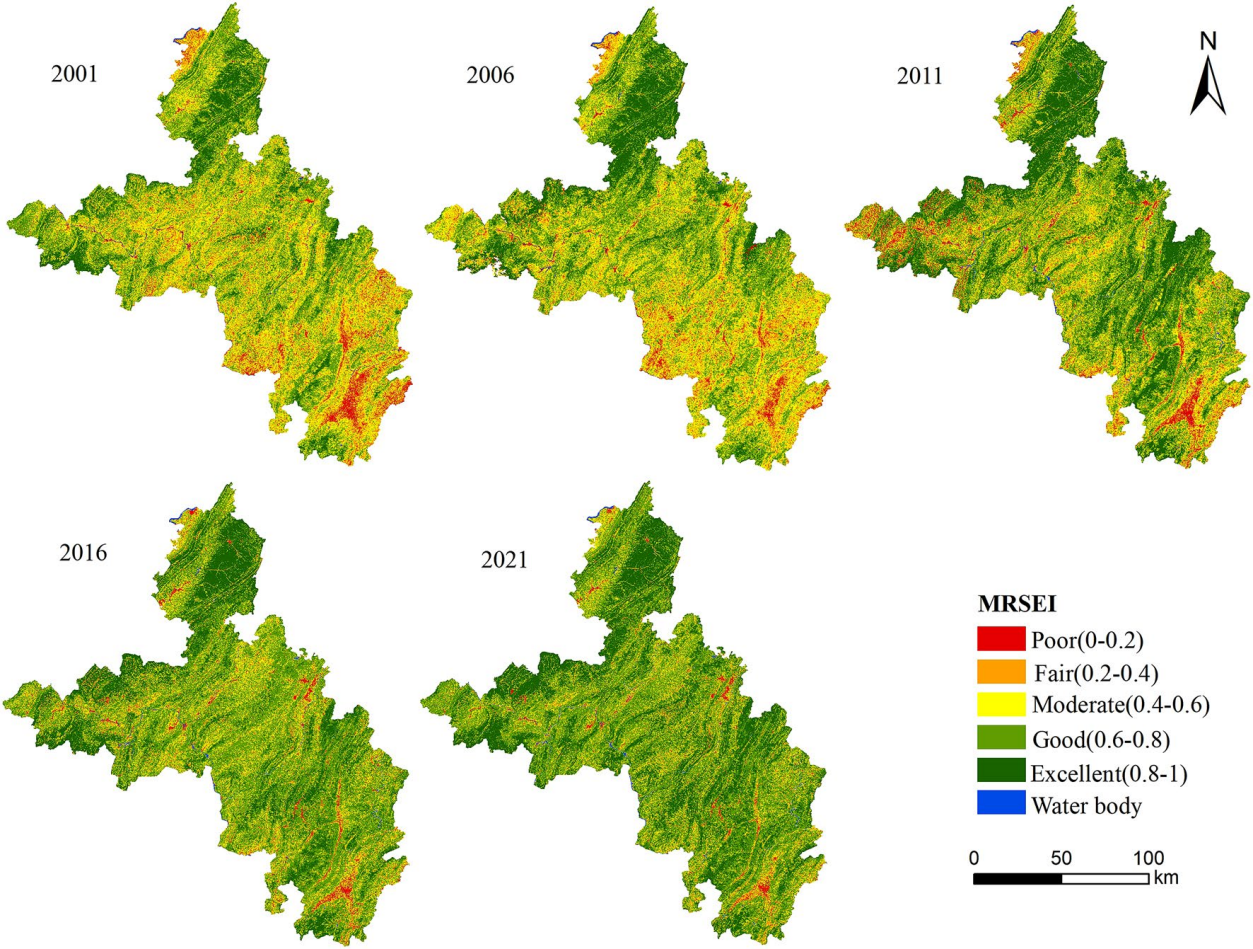
Distributions of the MRSEI grades. Map was generated by ArcGIS 10.2 (http://www.esri.com/software/arcgis/arcgis-for-desktop).

The spatial distribution in the MRSEI grades during the 20-year period was intuitively shown in Fig. 5. In terms of distribution characteristics, the ecological status in 2001 and 2006 was almost identical. Most of the regions with poor ecological quality were concentrated in the central, southeastern parts. The grades of ecological quality were mainly fair and good. In 2006, the ecological conditions in the central and southern parts were better than in 2001. By 2011, the overall situation had improved. Specifically, the ecological quality in the central and southeastern regions was improved obviously while the status in a few areas in the western part was worse than the situation in 2006. In 2016, the state in southeastern Chongqing continued to improve. The areas with poor ecological conditions significantly decreased, at the same time the situation in most regions was better than medium. By 2021, the ecological quality had entered a stage of sustained improvement and had gradually stabilized. Most of the ecological quality in the area was rated as good. Over the past two decades, the overall ecological environment in the region had continued to improve.

### Spatiotemporal changes in the ecological quality

**Figure 6:**
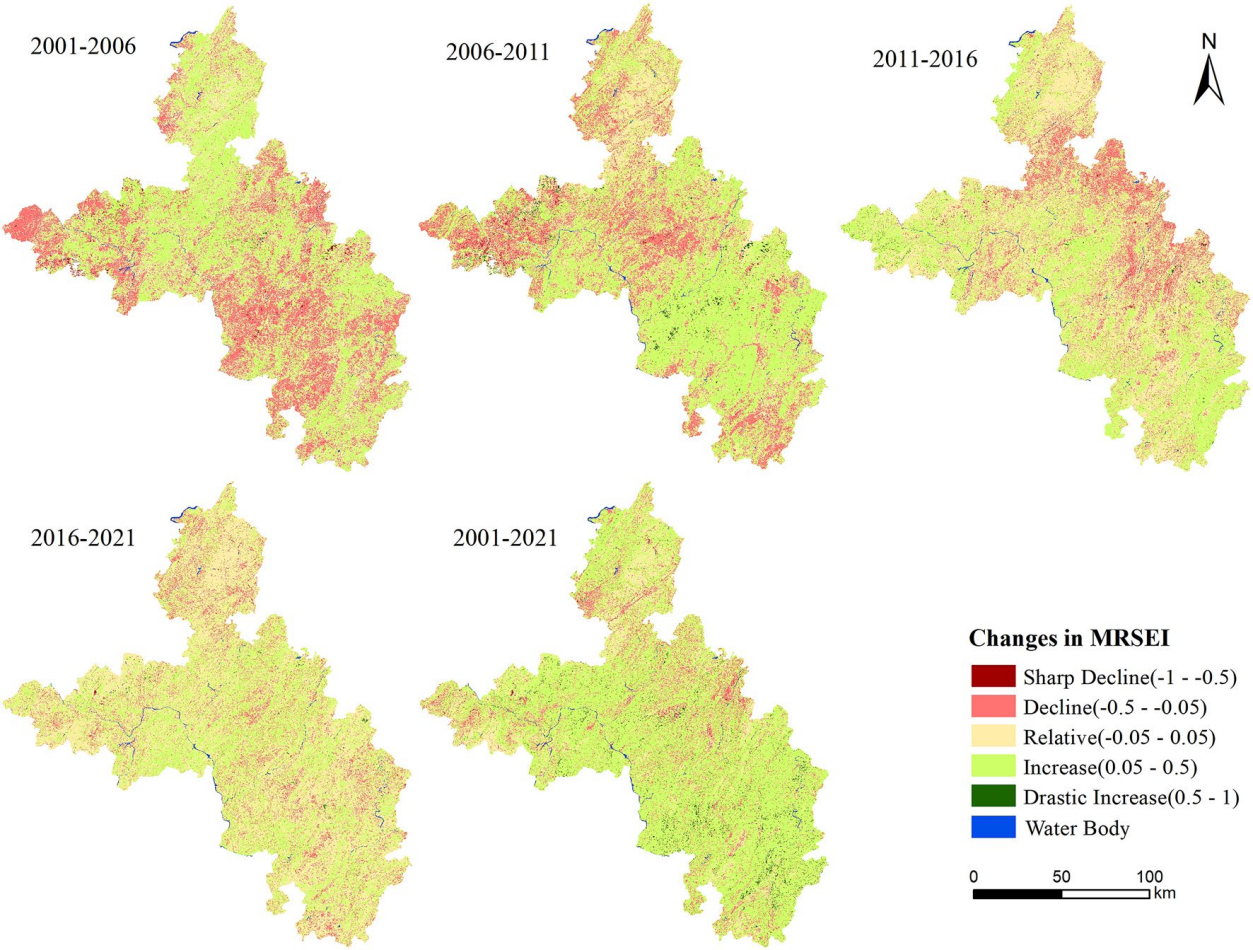
Distribution of the changes in the MRSEI grade. Map was generated by ArcGIS 10.2 (http://www.esri.com/software/arcgis/arcgis-for-desktop).

For investigating the variations in the MRSEI between 2001 and 2021, five descriptive labels were defined to distinguish the differences in MRSEI: sharp decline, decline, relatively stable, increase, and drastic increase. The difference in MRSEI for each period ranged from − 1 to 1, and each label represented a specific range of the MRSEI difference (Table 7, Fig. 6).

**Table 7:** Statistics of the changes in the MRSEI.

Level	2001–2006		2006–2011		2011–2016		2016–2021			2001–2021	
	Area	Pct	Area	Pct	Area	Pct	Area	Pct	Area		Pct
	(km^2^)	(%)	(km^2^)	(%)	(km^2^)	(%)	(km^2^)	(%)	(km^2^)		(%)
Drastic increase	14.834	0.078	120.425	0.634	28.254	0.149	6.833	0.036	221.315		1.166
Increase	6599.513	34.701	8139.882	42.854	7279.007	38.386	6535.238	34.431	12735.277		67.096
Relative stable	6479.888	34.072	6113.174	32.184	8220.882	43.353	10133.216	53.387	4143.672		21.831
Decline	5825.471	30.631	4571.394	24.067	3417.261	18.021	2289.260	12.061	1856.121		9.779
Sharp Decline	98.514	0.518	49.576	0.261	17.256	0.091	16.134	0.085	24.295		0.128

The area in which the condition of the ecological environment decreased from 2001 to 2006 was 5923.985 km^2^, while the improved area was 6614.347 km^2^. The overall situation was basically unchanged. In the second period, the area of ecological degradation was 4620.970 km^2^, accounting for 24.33% of the total area, which is lower than the previous period. The area of ecological improvement was 8260.326 km^2^, accounted for 43.49% of the total area, which was the highest proportion of the improved area among the four time periods. The ecological status has been greatly ameliorated. In the next 5 years, the area of ecological degradation was 3434.517 km^2^ (~ 18.11%), while there was an area of 7307.261 km^2^ (38.54%) become better. And the trend of ecological improvement was obvious. In the last period, the area of ecological degradation continued to decrease compared with the previous period. The ecological quality of most areas tended to be stable. The overall situation in the region continued to get better, but the improvement rate gradually slowed down during this time.

As can be seen from Fig. 6, the overall spatial distribution of the eco-environmental quality remained basically stable from 2001 to 2006. The improvement areas were mainly distributed in the northern and southeastern edge, the central parts, while the main degradation areas were concentrated in the western and southern regions. From 2006 to 2011, the status improved significantly. And the improved regions were relatively concentrated in the central and southern regions, and the deterioration occurred in the western part. The area in which the quality of the ecological environment improved was greater than the area in which it decreased. From 2011 to 2016, the situation continued to become better. The ecological quality in the western, southeastern edge, and central regions significantly improved while the condition in the eastern edge was decreased. From 2016 to 2021, the ecological quality in most of the regions was stable, and it was significantly improved in some southern and central regions. Besides, the areas with improved ecological environment quality were much larger than the areas of degradation.

In general, the condition of the ecological environment in southeastern Chongqing vastly improved during the past two decades. The ecological quality improved in nearly 70% of the study area. The improvement areas exhibited an increasing–decreasing–decreasing trend from 2001 to 2021. Obviously, the series of measures implemented for ecological governance have achieved remarkable results under strong promotion and vigorous implementation.

### Analysis of influencing factors on ecological quality

Considering the actual situation and based on previous studies, we selected appropriate indicators as the possible factors affecting the ecological environment. In view of the availability of data, we chose the EV and SP as topographical elements, MT, PR, RH and SH as climatic factors, LU, PD, GDP and NTL as elements of human activity. Then, we quantitatively analyzed the degree of the interpretation of each influencing factor to the spatial differentiation of the MRSEI using the geographical detector. Research on these factors could help to understand the factors influencing the spatial heterogeneity of the local ecological status.

The geographical detector revealed the influence of each detection factor on the MRESI differentiation by calculating the q value (Table 8). In 2001, the q values of the MT, EV, and PD were the top three, reaching 0.418, 0.348 and 0.307, respectively. In 2006, the q values of the SP, PR and RH were 0.406, 0.397 and 0.36, respectively, so they were the main causes influencing the ecological status during this period. And in 2011, the dominant factors were the EV, RH, and MT. In the next period, the EV, NTL and PR were the primary factors affecting the ecological quality. In 2021, the foremost factors influencing the ecological condition were the SP, EV and GDP. Overall, from 2001 to 2021, the dominant factors affecting the MRSEI were the EV, SP, PR and LU based on their mean q values. In contrast, the SH, GDP, NTL and PD had little influence on the MRSEI. In addition, the q values of each detection factor from 2000 to 2021 exhibited varying degrees of fluctuation. The possible reason for the different degrees of variation in the q values was that the interannual variations in the detection factors directly or indirectly affected the surface–water-heat status and vegetation growth. Or because there were other natural factors that had a great effect on the ecological environment, resulting in spatial differentiation of the ecological environment over many years.

**Table 8:** q values of the detection factors.

Driving factors	q statistic value					
	2001	2006	2011	2016	2021	Mean
EV	0.348*	0.170*	0.388*	0.350*	0.269*	0.305*
SP	0.297*	0.406*	0.135*	0.210*	0.326*	0.275*
PR	0.063*	0.397*	0.288*	0.252*	0.133*	0.227*
MT	0.418*	0.112*	0.304*	0.094*	0.101*	0.206*
RH	0.083*	0.360*	0.327*	0.103*	0.081*	0.191*
SH	0.055*	0.019*	0.105*	0.085*	0.015*	0.056*
LU	0.226*	0.152*	0.309*	0.229*	0.209*	0.225*
PD	0.307*	0.202*	0.097*	0.148*	0.064*	0.164*
GDP	0.110*	0.071*	0.097*	0.045*	0.217*	0.108*
NTL	0.098*	0.091*	0.160*	0.339*	0.049*	0.148*

*Indicates significance at 0.01 level.

## Discussion

### Comparison of RSEI, MRSEI and EI

When constructing the MRSEI, this study also extracted the RSEI of the study area. The PCA was carried out on the superimposed layers of the four indexes of NDVI, WET, NDBSI and LST. The results showed that in 2001 and 2006, the contribution rates of the eigenvalues of the first principal component were 59.60%, 62.10%, respectively. After adding the rocky desertification degree index, the contribution rate of the eigenvalues of the first principal component of the PCA results in each period increased. Therefore, MRSEI can better integrate the characteristics of each index component. Taking 2021 with the highest contribution rate of the first principal component as an example, the RSEI in 2021 was constructed. And at the same time, the EI in 2021 was constructed. By analyzing the consistency of RSEI, MRSEI and EI, the rationality of MRSEI was verified.

**Figure 7:**
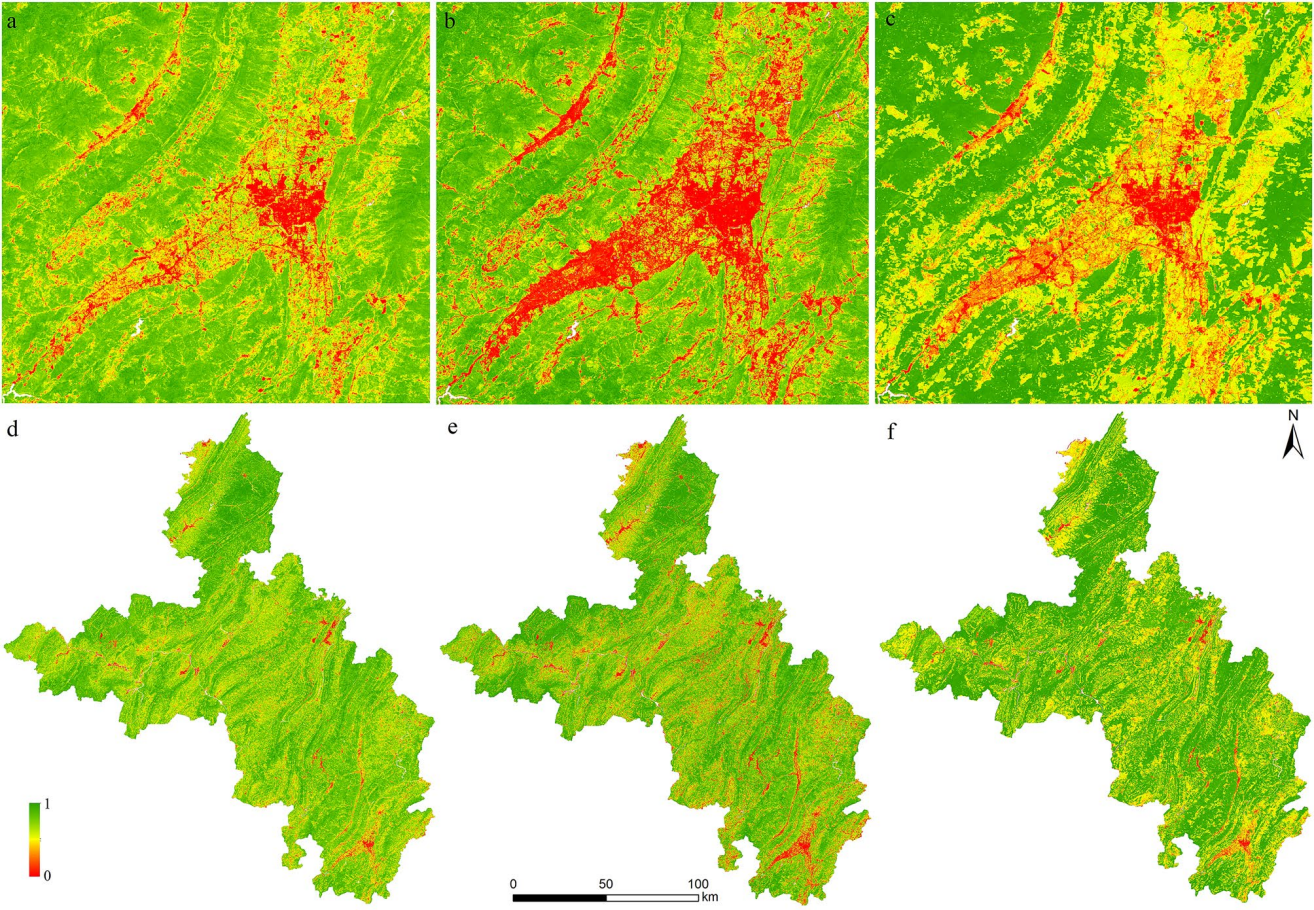
Comparison of RSEI, MRSEI and EI in 2021: a typical ecologically poor area (**a**) in RSEI (**d**), the same area (**b**) in MRSEI (**e**) and the same area(**c**) in EI (**f**). Map was generated by ArcGIS 10.2 (http://www.esri.com/software/arcgis/arcgis-for-desktop).

**Figure 8:**
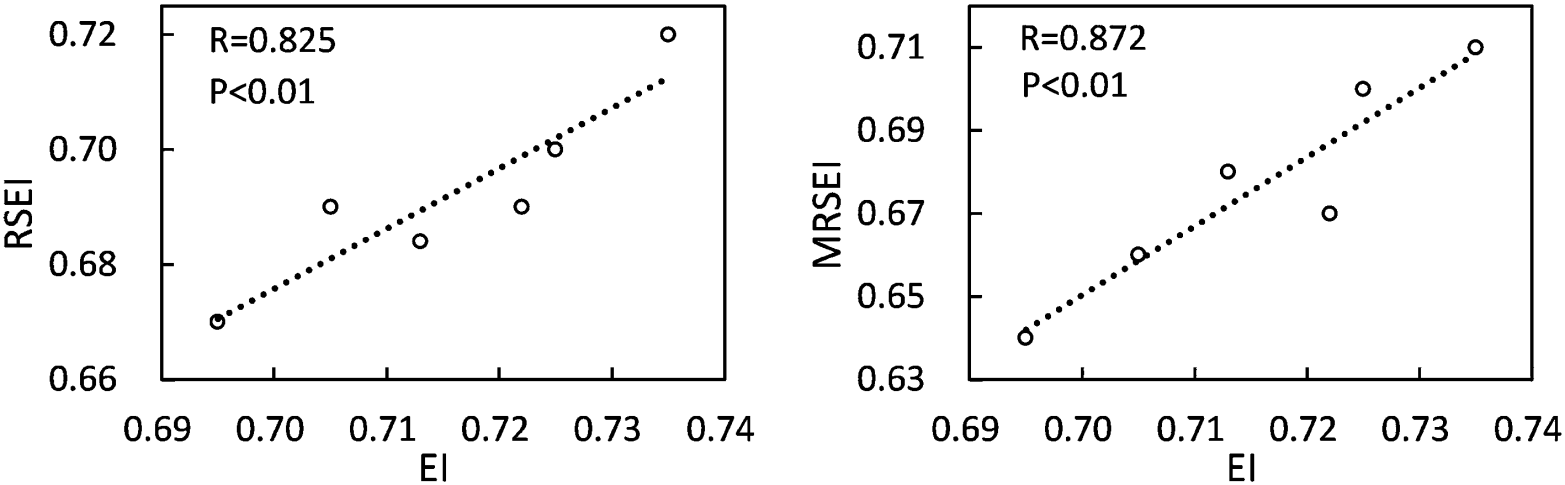
Correlation coefficient between RSEI/MRSEI and EI.

The distribution and changing trends of RSEI, MRSEI and EI in the study area were highly consistent, but in the same area, the ecological environment reflected by EI was generally better than that reflected by RSEI and MRSEI (Fig. 7). Since EI was used points to interpolate to the surface, it focused on the changing trend of ecology, but cannot clearly reflect the local details. Therefore, the mean values of MRSEI and RSEI of each district and county in the study area were obtained, which represented the overall ecological quality of the area. The obtained results were then correlated with the EI of each district and county (Fig. 8). It can be seen from Fig. 8 that the correlation coefficient between RSEI and EI and the correlation coefficient between MRSEI and EI were 0.825 and 0.872 (P < 0.01), respectively. Although both RSEI and MRSEI can reflect the quality of the ecological environment, the correlation between MRSEI and EI was higher than that between RSEI and EI. Therefore, MRSEI was superior to RSEI in ecological quality monitoring and quantitative description in the study area.

### Limitations and further study

There are still some limitations should be focus on although the evolutionary results of the ecological quality in the target area from 2001 to 2021 were obtained.

Firstly, due to the lots of clouds and fog in the study area, the images used are limited. For those that did not meet the research requirements, images from the same period in adjacent years were selected for replacement. The MRSEI constructed after replacing the images did not fully and accurately represent the actual situation in the target year. Although the results could reflect the evolution of the ecological status of the target area in the past two decades, they could not reflect the changes in each year. The study period of this study was also not continuous enough. Secondly, in the construction of the MRSEI, the water bodies needed to be masked in this study. Therefore, only the terrestrial ecological environment was considered, so the results fail to fully show the effect of water on the ecological environment. Finally, the analysis results of the influencing factors on the ecological quality status obtained by the geographic detector model cannot be visualized in space by region, which weakened the integrity of the analysis to a certain extent. And it was found that although the influencing factors selected in this study passed the significance test, and they had a certain connection with the MRSEI. But due to the availability of data, some influencing factors had a low independent interpretation ability for the MRSEI.

In future research, other images can be combined to overcome the limitations imposed by the data discontinuity and then, the evolution in the regional ecological environment status can be analyzed year by year. And it is possible to explore the effects of natural factors such as soil and lithology, and human factors such as agricultural planting systems, land consolidation projects, and special land use planning on ecological quality through further data collection. In addition, more appropriate methods can be selected to analyze the factors influencing the ecological conditions, and their degrees of influence can be visualized to make the ecological quality protection measures more effective and targeted.

### Suitability of this research method in other regions

The study area is located in the karst area of southwestern China, which is a typical rocky desertification area. Rocky desertification is widely distributed in this region, and the problem of rocky desertification has become the main obstacle to local ecological environment construction and sustainable development. In view of the rocky desertification phenomenon, this study constructed MRSEI on the basis of RSEI and combined the degree of rocky desertification to monitor and analyze the ecological status in southeastern Chongqing from 2001 to 2021. At the same time, the comprehensiveness and rationality of MRSEI have been verified. The method adopted in this research can provide a reference for the assessment of ecological quality in karst areas and it would be a good attempt to apply the method to the corresponding research in other similar areas.

## Conclusions

From the correlation analysis between the MRSEI and each index component, it can be seen that MRSEI has high comprehensiveness and representativeness for greenness, humidity, heat, dryness and rocky desertification degree. And to a certain extent, the rationality of MRSEI is proved by the comparative analysis and correlation analysis with the EI. From the effect of the component indicators: the NDVI and WET could give some positive effects on the MRSEI, while the LST, NDBSI, and RI had negative impacts. And RI had a vital influence on the ecology, which was an important reason for affecting the ecological conditions.

From 2001 to 2021, the analysis revealed that the MRSEI in the study area increased from 0.561 to 0.678, indicating that the overall situation in the ecological environment had a significant increase. Since 2006, rocky desertification control measures have been vigorously implemented in the study area, and the RI has decreased by 27.1%. In the last two decades, the ecological status had greatly improved. The proportion of the improved area was nearly 70% of the entire region, and it exhibited an increasing–decreasing–decreasing trend. Recently, the situation of the ecological environment has continued to improve, but the improvement trend has gradually flattened.

Furthermore, the main factors influencing the ecological quality in southeast Chongqing were the elevation, slope, monthly average precipitation, and land use pattern. In future studies, it would be better to further develop the regional ecological quality evaluation system and collect data on additional influencing factors to research on the ecological status and the reasons for its changes.

## Data Availability

The data that support the findings of this study are available. These data were derived from the following resources available in the public domain: the remote sensing data were obtained from the United States Geological Survey (USGS, https://earthexplorer.usgs.gov/. The elevation data was provided by the official website of the United States Geological Survey (USGS, https://earthexplorer.usgs.gov/). The meteorological data, including the monthly average temperature (MT), monthly mean precipitation (PR), monthly even relative humidity (RH), and monthly total sunshine hours (SH) from May to September of the target year, were got from the China Meteorological Data Network (http://data.cma.cn/). The socioeconomic data, including the population density (PD) and gross domestic product (GDP), were obtained from the statistical yearbooks of each district and county in the study area. The nighttime light (NTL) data were obtained from the National Oceanic and Atmospheric Administration (NOAA, https://www.noaa.gov/). The statistical data and monitoring data of each evaluation index used to construct the EI come from the statistical yearbooks, water resources bulletin and soil and water conservation bulletin of each district and county.
